# Detection of strong attractors in social media networks

**DOI:** 10.1186/s40649-016-0036-9

**Published:** 2016-12-07

**Authors:** Ziyaad Qasem, Marc Jansen, Tobias Hecking, H. Ulrich Hoppe

**Affiliations:** 1 Computer Science Institute, University of Applied Science Ruhr West, Lützowstraße 5, 46236 Bottrop, Germany; 2 Department of Computer Science and Applied Cognitive Science, University of Duisburg-Essen, Lotharstraße 63, 47057 Duisburg, Germany

**Keywords:** Detection of attractors, Social media networks, Twitter, Asterisk, Information diffusion, Independent cascade model

## Abstract

**Background:**

Detection of influential actors in social media such as Twitter or Facebook plays an important role for improving the quality and efficiency of work and services in many fields such as education and marketing.

**Methods:**

The work described here aims to introduce a new approach that characterizes the influence of actors by the strength of attracting new active members into a networked community. We present a model of influence of an actor that is based on the attractiveness of the actor in terms of the number of other new actors with which he or she has established relations over time.

**Results:**

We have used this concept and measure of influence to determine optimal seeds in a simulation of influence maximization using two empirically collected social networks for the underlying graphs.

**Conclusions:**

Our empirical results on the datasets demonstrate that our measure stands out as a useful measure to define the attractors comparing to the other influence measures.

## Introduction

Social media have become an important information resource to gain insights into and acquire knowledge about a wide variety of more or less numerous communities interacting through the internet. Moreover, applying analytic techniques to social media data can support better informed decision-making processes in numerous fields, such as marketing, politics and education. One prominent aspect of such analytics is the characterization and detection of influential actors in social networks. There are several studies on social media which have suggested different approaches and specific measures to solve the problem of influential actor detection.

In this paper, we elaborate on a new approach for the detection of influential actors which is based on quantifying the contribution of this actor to increasing the size of the network by attracting new active members of the specific subcommunity [1]. In comparison to weighted or unweighted indegree measures, our new measure would only count those neighbors who were new to the network when the relationship to the actor in focus was first established. In other words, an actor who has a high value in terms of this measure has been an important "target” node for the attraction of new members to the network and this for increasing the overall size of the network. A formal specification of this property (referred to as "*T* measure”) is given in the first part of the paper.

Our approach can be applied to social networks in which timestamps are attached to edges connecting to actors. In the evaluation section of this paper, we apply our approach first to dataset from the Asterisk open source software developer community (a relatively small community with less than 1400 members and much less active actors) to test whether the influential actors who are already known from the Asterisk mailing list can be also identified using our approach. Second, we use a bigger dataset based on Twitter communication around #EndTaizSiege and #coup_suffocates_Taiz (related to recent events in Yemen). Here, we compare our approach with other standard measures such as indegree, and betweeness in terms of how good these measures are if used to generate seeds for an independent cascade diffusion process. The objective of studying our *T* measure in the field of information diffusion is to show that *T* measure is effective to define influential actors who are effective in attracting others to become active in a specific community.

The rest of the paper is organized as follows: "Literature review" section presents related research. An overview of our proposed approach is given in "Approach" section, which also provides the basic formal definitions. "Implementation" section introduces the concept, followed by the description of our datasets and the experimental results in "Experimental results" section. "Information diffusion and *T* measure" and "Simulation of attraction processes with time-respecting paths" sections deal with the performance of our approach in the influence maximization problem. Finally, conclusions are drawn and an outlook for further research is described in "Conclusion" section.

## Literature review

In this section, we review studies of influence in social media such as Twitter and remind the concept of information diffusion and its relation with the type of influence on which our approach is based.

### Influence in social media

In the field of social media analysis, there exists a large body of research on modeling and measuring influence and on detecting influential actors. Here, social networking platforms such as Twitter are of special interest. However, regarding the manifestation and identification there are still open questions. Researchers have studied influence in social media networks, and many approaches rank users according to their influence.

Leavitt et al. [2] employ four features to evaluate influence, which are replies, retweets, mentions, and number of followers. They support statistical results related to these measures, but do not present a global influence measure based on all the suggested criteria. In the work of Cha et al. [3], it could be shown that employing different measures can lead to completely different results when it comes to the task of ranking users according to their importance. Results were presented based on Twitter data and three different measures of influence, namely indegree (number of followers of an actor), retweets (number of retweets containing one’s actor name), and mentions (number of mentions containing one’s actor name). They presented an in-depth comparison of these measures with the conclusion that different measures can be used to identify different types of influential actors. Indegree tends to be highest for news sites and celebrities, and thus, is suited to model popularity. However, the number of followers (indegree) does necessarily go along with a high number of retweets or mentions. The number of retweets is highest for information aggregation services and the number of mentions for celebrities. Consequently, the way in which a network is extracted from social media content and the measure of influence should be considered carefully with respect to the roles and type of influence one aims to uncover. Azaza et al. [4] proposed a new influence assessment approach depending on belief theory to combine different types of influence markers on Twitter such as retweets, mentions, and replies. They used Twitter dataset of European Election 2014 and deduced the top influential candidates. In our approach, we depend on the retweet relation as a marker to attract others to become active in a specific community in which a specific topic is dealt. As well as, a retweet relation can be understood as a form of information diffusion and as a means of participating in an event in social media [5].

Other researches propose to define influential actors based on link analysis. Twitter User Rank (TURank) [6] is an algorithm which utilizes ranking algorithms to define authoritative actors on Twitter, based on link analysis. TURank introduces actor–tweet graph where nodes are actors and tweets, and edges are follow and retweet relationships. TwitterRank [7] extended PageRank algorithm to measure influential actors in Twitter based on link structure and topical similarity.

Apart from the pure network information, influence can also be modeled additionally taking into account the actions of actors (e.g. on Flickr [8]), similarity of actors [9], and produced content associated with each actor [10].

Our work aims for a clear formulation of social influence and a methodology to produce an exact ranking of the actors according to the definition. In concrete, we provide a new type of influence in online social network to emphasize on those actors who attract many outsiders to join the own community in which a specific topic is dealt. For example, in Twitter those actors spawn many retweets on a certain topic from people who have no previous contributions on that topic. This new type of influence led us to propose a new approach to detect those actors, and compare the results with other standard measures.

### Information diffusion

Influence is often related to information diffusion in a network. Information diffusion is the process by which a new idea or innovation spread over the networks by the means of connection among the social network actors [11]. Especially in social media, influential actors can control the diffusion of information through the network to some extent.

There are numerous research on the information diffusion over social network. For instance, Gruhl et al. [12] studied and modeled the dynamic of information diffusion on blogsspace environment. Yang et al. [13] proposed a model to capture the attribute of information diffusion which are related to speed, scale, and range. With spreading of information diffusion models and their variations, Vallet et al. [14] used graph rewriting to compare the different information diffusion models.

Widely used information diffusion models are the independent cascade (IC) [15, 16] and the linear threshold (LT) [17]. The two models describe different aspects of influence diffusion. IC model focuses on influence among neighbors on social network, and LT model focuses on the threshold behavior in influence diffusion [18].

Kempe et al. [19] proposed to use the IC and LT models to solve the influence maximization problem which asks for a set of actors whose aggregated influence in the social network is maximized, whereas Pei et al. [20] provided strategies to search for spreaders based on the following of information flow rather than simulating the spreading dynamics (modeled_dependent results). The study of [19] was followed by several research on the same problem (e.g. [18, 21, 22]). Furthermore, the features of identifying spreaders measures using independent interaction and threshold models through empirical diffusion data from LiveJournal are discussed in [23]. Morone et al. [24] proposed to map the problem of influence maximization in complex networks onto optimal percolation using Collective Influence (CI) algorithm.

In this paper, we evaluated the performance of our measure *T* in the information diffusion maximization problem by selected sets of top actors based on *T* measure and other sets which are defined by other standard measures. The advantage of our measure is to consider a new type of influence which refers to actors who attract others to be active in a particular community. Thus, we use the IC model to evaluate the performance of our measure comparing with other standard measures.

## Approach

Our approach is based on this premise: the more a certain actor (Actor *a*) attracts new actors, the more actor *a* is important to the social network. Thus, in this approach we tried to evaluate the attractiveness value of social media actor which leads us to detect the attractors.

In this section, we will provide some definitions for special terms that help to provide a profound methodology in presenting our approach. This approach is based mainly on the decomposition of data collected from a given social network according to the time period of collection. Let us refer to that period by the term *P*-period. For instance, if the *P*-period of a given social network is 30 days, the social network data collection took 30 days.

### **Definition 1**

(*P*-*period*) *P*-period is a time duration of the data collection process from social networks.

In this paper, the social networks’ data are depicted by a graph representation. To distinguish this graph in any context, it is defined under the name *P*-graph. Thus, we can say that our approach is based on the decomposition of the *P*-graph into subgraphs depending on the *P*-period.

### **Definition 2**

(*P*-*graph*) *P*-graph is a graph constructed from social network data which have been collected during *P*-period. Thus, the collected graph during *P*-period is described by *P*-graph *G*(*V*, *E*), where

- *V* is the set of all actors who joined the community during *P*-period.
- *E* is the set of all connections that have been established between the actors *V* during *P*-period.

Decomposition of a *P*-graph based on *P*-period requires decomposition of the *P*-period into slices of time so that every subgraph is related to a slice. In our approach, we refer to each slice as *P*-slice.

### **Definition 3**

(*P*-*slice*) *P*-slice is a time slice of *P*-period.

If all *P*-slices are equidistant, then we define a special case of *P*-slice as *EP*-slice. For example, let *P*-period be 30 days and the number of slices be 5 *EP*-slices. Then, the value of each *EP*-slice will be as in Table 1. We notice that each *P*-slice is included in the later ones.

**Table 1:** *EP*-slice values for *P*-period of 30 days

*EP*-slice	Value
$s_1$	6
$s_2$	12
$s_3$	18
$s_4$	24
$s_5$	30

### **Definition 4**

(*EP*-*slice*) *EP*-slice is a *P*-slice such that all *P*-slices are equidistant.

To facilitate the definition of subgraphs of this approach, we will define some terms related to actors according to *P*-slices.

### **Definition 5**

(*P*-actors) Let $s_1,s_2,\ldots s_n$ be the *P*-slices. For every *i* such that $0 < i \le n$, the *P*-actors $A_i$ is a set of all actors that joined the social network between 0 and $s_i$.

### **Definition 6**

($P_s$-*actors*) Let $s_1,s_2,\ldots s_n$ be the *P*-slices. For every *i* such that $0 < i \le n$, the $P_s$-actors $A_{s_i}$ are a set of all actors that joined the social network between the *P*-slices $s_{i-1}$ and $s_i$.

**Fig. 1:**
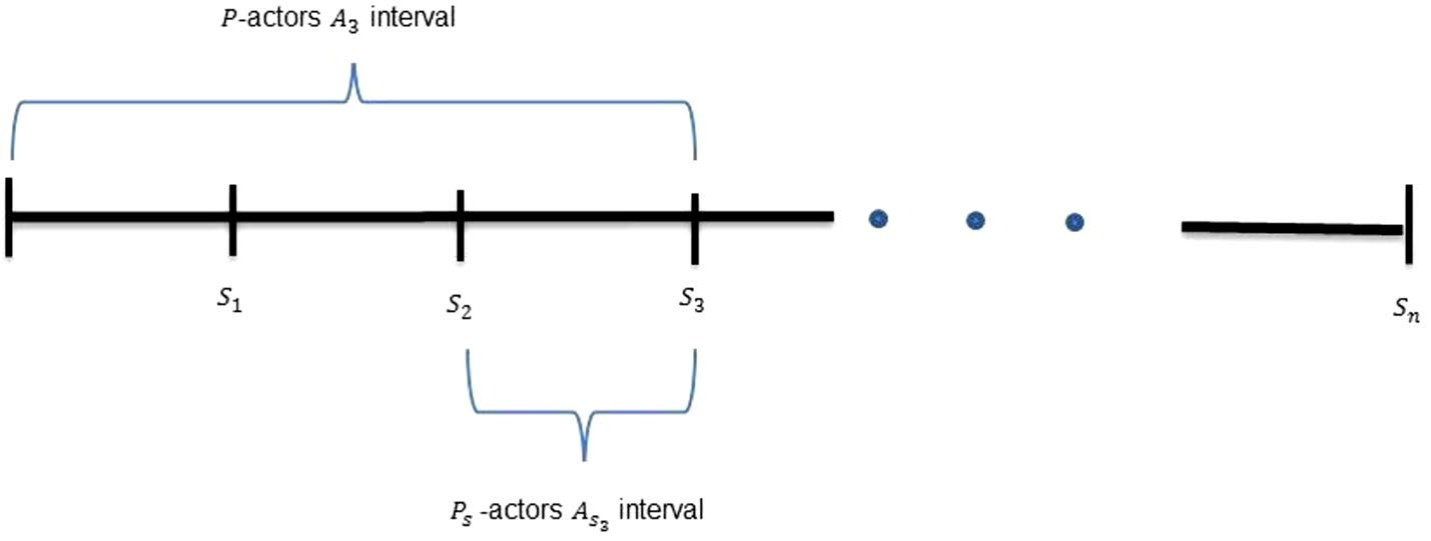
*P*-actors and $P_s$-actors with respect to *P*-slices

Figure 1 shows how the *P*-actors and $P_s$-actors are taken with respect to *P*-slice in our approach. The figure displays the *P*-actors $A_3$ and $P_s$-actors $A_{s_3}$ as an example. $A_3$ joined the social network between *P*-slices $s_0$ and $s_3$ whereas $A_{s_3}$ joined between *P*-slices $s_2$ and $s_3$.

After discussing the terms mentioned above, now it is easy to provide the definitions for the different types of subgraphs which will be used in this approach with. These definitions will be helpful on our way to reach the goal of this approach.

### **Definition 7**

(*P*-*subgraph*) *P*-subgraph $G_i(A_i,E_i)$ is a subgraph of *P*-graph *G* which is aggregated until *P*-slice *i*. Thus, the aggregated subgraph until *P*-slice *i* is described by the *P*-subgraph $G_i(A_i,E_i)$, where

- $A_i$ is the *P*-actors $A_i$.
- $$E_i= \{(a,b) : a,b\in A_i\}$$

By this, we focus on the connections by which the actors attracted the new actors; hence, we can easily measure the actors’ attractiveness. The next definition will discuss this issue in formal way.

### **Definition 8**

(*S*-*subgraph*) The *i*th *S*-subgraph $S_i(A_i,E_{s_i})$ is a subgraph of the *P*-subgraph $G_i(A_i,E_i)$ such that

- $A_i$ is the *P*-actors $A_i$.
- $$E_{si}= \{(a,b) : a\in A_{i-1} \ {\rm and} \ b\in A_{s_i}\} \ \cap E_i$$

**Fig. 2:**
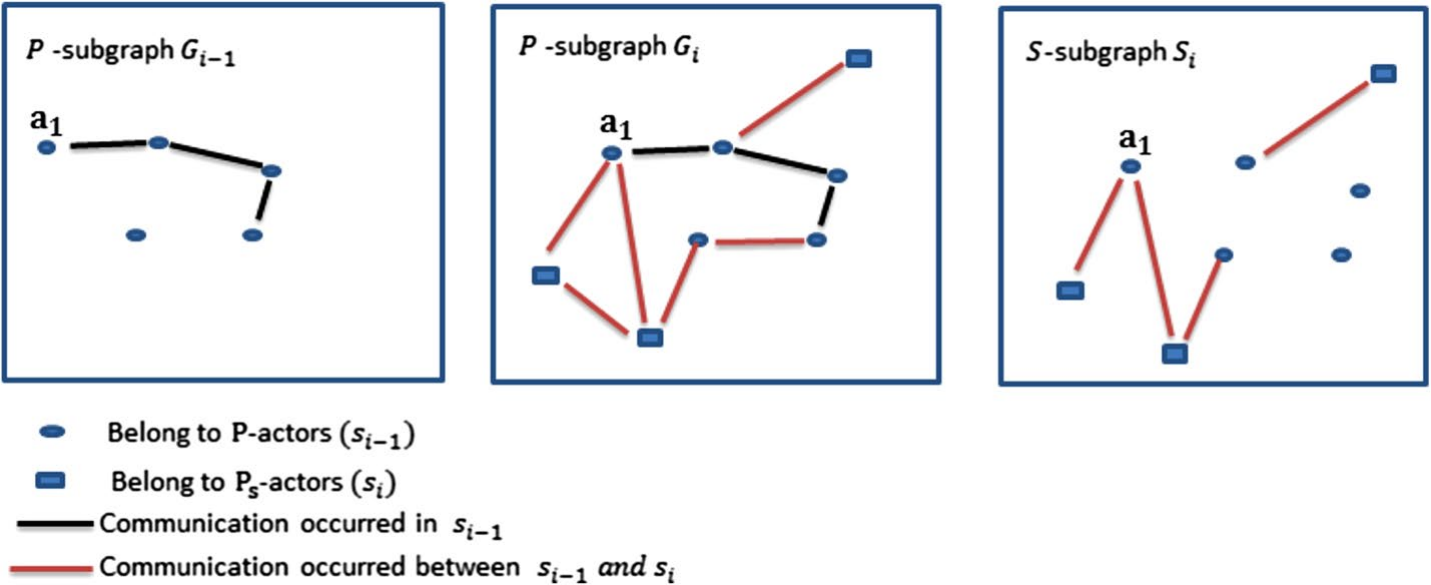
*P*-subgraphs $G_{i-1}$ and $G_{i}$, and *S*-subgraph $S_{i}$

From Definition 8, we notice that *S*-subgraph $S_i$ contains the new connections by which the new actors $A_{s_i}$ joined the network. The number of these connections refers to the attractiveness value of the actors $A_{i-1}$. Later in the implementation section, Definition 8 is used to facilitate the calculation of the attractiveness value *T*. Figure 2 shows the difference between *P*-subgraph and *S*-subgraph in our approach where *n* is the number of *P*-slices and $1<i\le n$. *P*-subgraph $G_{i-1}$ is the *P*-subgraph of the *P*-slice $s_{i-1}$, and *P*-subgraph $G_{i}$ and *S*-subgraph $S_{i}$ are of the *P*-slice $s_{i}$.

What if the *P*-graph is a directed graph? The *P*-subgraph would be directed with the same properties of *P*-subgraph in Definition 7; however, the definition of the *S*-subgraph would be slightly different.

### **Definition 9**

(*Directed S*-*subgraph*) The *i*th directed *S*-subgraph $S_i(A_i,E_{s_i})$ is a subgraph of the directed *P*-subgraph $G_i(A_i,E_i)$ such that

- $A_i$ is the *P*-actors $A_i$.
- $$E_{s_i}= \{(a,b) : ( \ a\in A_{i-1} \ and \ b\in A_{s_i} \ ) \ or \ ( \ b\in A_{i-1} \ and \ a\in A_{s_i} \ ) \} \ \cap E_i$$

**Fig. 3:**
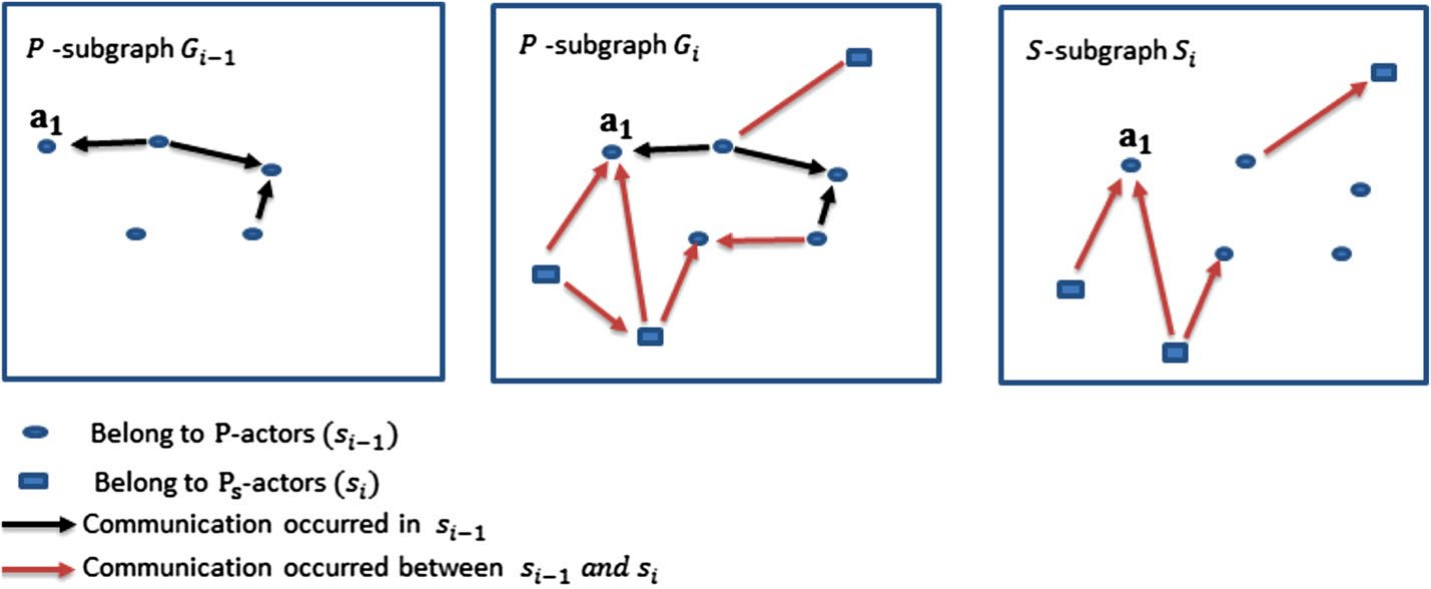
Directed *P*-subgraphs $G_{i-1}$ and $G_{i}$, and directed *S*-subgraph $S_{i}$

In Fig. 3, the directed *P*-subgraph and *S*-subgraph are shown where *n* is the number of *P*-slices and $1<i\le n$. The directed *P*-subgraph $G_{i-1}$ is the *P*-subgraph the *P*-slice $s_{i-1}$, and the directed *P*-subgraph $G_{i}$ and *S*-subgraph $S_{i}$ are of the *P*-slice $s_{i}$.

In the next section, we will introduce the implementation of our approach to evaluate the attractiveness value of each actor in online social media.

## Implementation

According to the *P*-slices, the *P*-graph in this approach is decomposed into *n* *P*-subgraphs $G_1,G_2,\ldots G_n$ and $(n-1)$ *S*-subgraphs $S_{2},S_{3},\ldots S_{n}$ where *n* is the number of *P*-slices. To evaluate the attractiveness value of each actor in each P-subgraph, we use the formula in next definition.

### **Definition 10**

(*Attractiveness value T*) Let $s_1,s_2,\ldots s_n$ be the *P*-slices. For every *i* such that $0< i < n$, the attractiveness value of an actor *a* in *P*-subgraph $G_i$ is given by the expression:

1 $$\begin{aligned} T(a_{G_i})= \left\{ \begin{array}{ll} o &{} \text{ if } a \notin A_i \\ \frac{\text{deg}(a_{S_{(i+1)}})}{|A_{s(i+1)}|} &{} \text{ if } a \in A_i \end{array} \right. \end{aligned}$$

where $T(a_{G_i})$ is the attractiveness value of actor *a* in *P*-subgraph $G_i$, ${\text deg}(a_{S_{(i+1)}})$ is the degree of the same actor but in *S*-subgraph $S_{{(i+1)}}$, and $A_s{(i+1)}$ is the $P_s$-actors in *S*-subgraph $S_{(i+1)}$.

From Fig. 2, we notice that the attractiveness value of the actor $a_1$ in *P*-subgraph $G_{i-1}$ is equal to 2/3 which is resulted from his/her degree in *S*-subgraph $S_i$ divided by number of $A_{s_i}$.

Now, we provide the way by which the new measure of attractiveness can be evaluated. Let us call the new measure by *T*, and it is evaluated as follows:

### **Definition 11**

(*Measure T*) Let $s_1,s_2,\ldots s_n$ be the *P*-slices. For every *i* such that $0< i < n$, the *T* value $T(a_G)$ of an actor *a* in *P*-graph *G* is given by the expression:

2 $$\begin{aligned} T(a_G)={\sum \limits _{i=1}^{n-1} T(a_{G_i})} \end{aligned}$$

where $T(a_{G_i})$ is evaluated relating to Eq. 1. To normalize the value of *T* measure to be between 0 and 1, we will divide Eq. 2 by $(n-1)$ as follows:

3 $$\begin{aligned} T_n(a_G)=\frac{\sum \limits _{i=1}^{n-1} T(a_{G_i})}{n-1} \end{aligned}$$

**Fig. 4:**
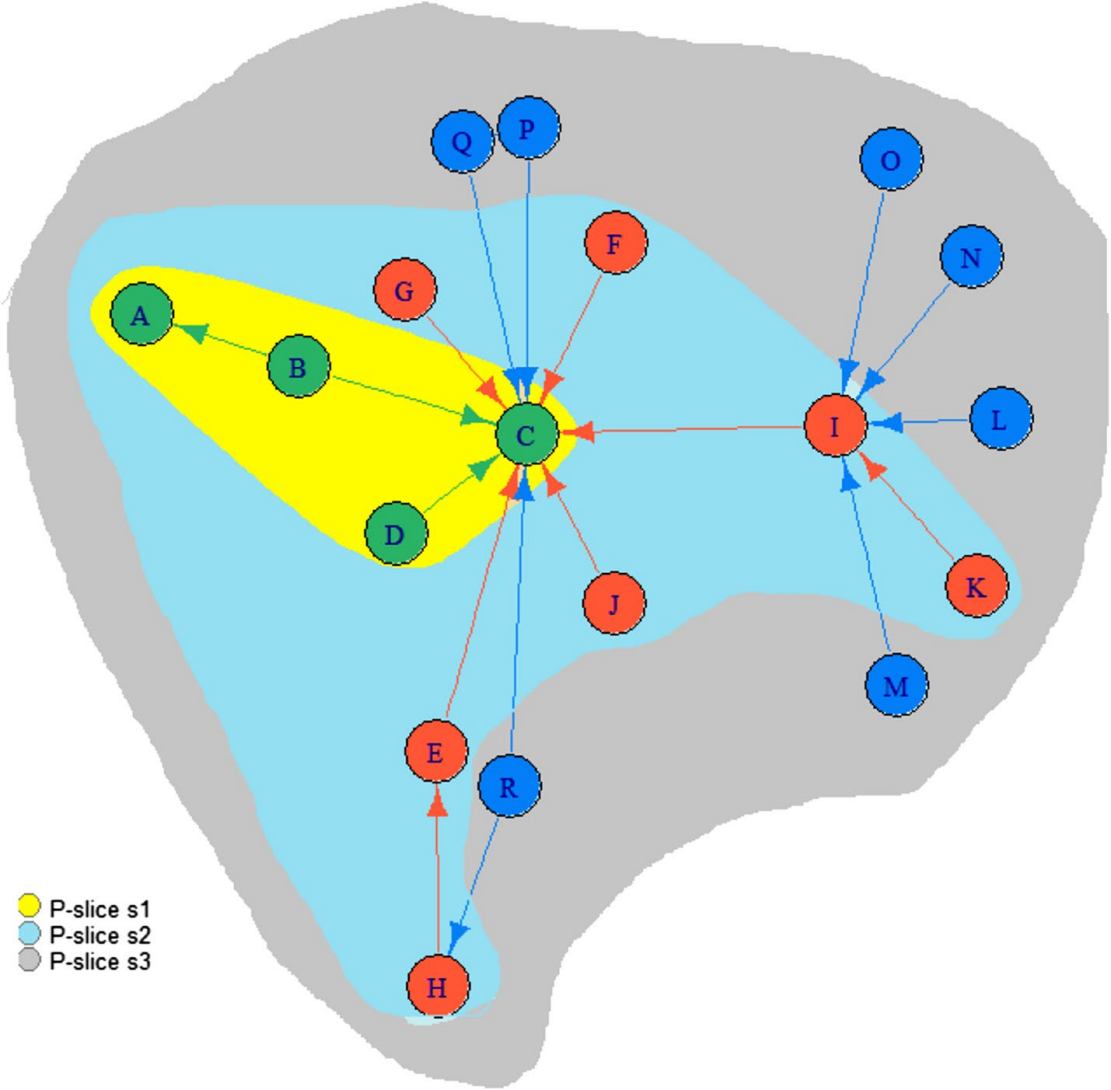
Toy example: *P*-graph *G* with three *P*-slices

Figure 4 shows an example of an *P*-graph *G* with three *P*-slices. With respect to our approach definitions, we can expose that we have three *P*-subgraphs and two *S*-subgraphs. From Fig. 4, we can get for instance:

- $A_{s_2}$ which is the set of the $P_s$-actors *E*, *F*, *G*, *H*, *I*, *J*, and *K*.
- *P*-subgraph $$G_2(A_2,E_2)$$ where
  - $A_2$ is the set of the *P*-actors *A*, *B*, *C*, *D*, *E*, *F*, *G*, *H*, *I*, *J*, and *K*.
  - $E_2$ is the set of the connections (*B*, *A*), (*B*, *C*), (*D*, *C*), (*E*, *C*), (*H*, *E*), (*G*, *C*), (*F*, *C*), (*I*, *C*), and (*K*, *I*).
- *S*-subgraph $$S_2(A_2,E_{s_2})$$ where
  - $E_{S_2}$ is the set of the connections (*E*, *C*), (*H*, *E*), (*G*, *C*), (*F*, *C*), (*I*, *C*), and (*K*, *I*).
  To calculate the attractiveness value of the actor *C* in the whole *P*-graph *G*, we have to calculate

- $T(C_{G_1})$ which equals the indegree value of the actor *C* in the *S*-subgraph $S_2$. In this case, it equals 5. In normalized form, we evaluate also the number of $P_s$-actors $A_{s_2}$ which equals 7. Thus, $T(C_{G_1})$ equals 5/7
- $T(C_{G_2})$ which equals the indegree value of the actor *C* in the *S*-subgraph $S_3$. In this case, it equals 3. In normalized form, $T(C_{G_2})$ equals 3/6, where 6 is the number of $P_s$-actors $A_{s_3}$.

With respect to Eq. 2, the *T* value of the actor *C* in the whole *P*-graph *G* equals $T(C_{G_1})$ plus $T(C_{G_2})$ which is 1.214.

In this section, we will describe the type of our dataset, and the characteristic of each type. Furthermore, the experimental results on the different dataset will be discussed in this section.

### Evaluation strategy

**Fig. 5:**
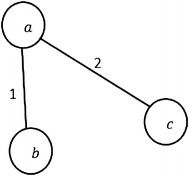
An example of the graph representation for Asterisk dataset

Our approach has been applied to three different datasets. First, we chose the open source software development project Asterisk. Here, the dataset originated from the communications in the developer mailing lists during 2006 and 2007. The Asterisk dataset contains 13,542 messages and 4694 threads that were discussed by 1324 developers. Two actors are linked if they participated in the same mailing thread. Figure 5 shows an example of an actor *a* participating once in the same mailing thread with actor *b* and having shared two mailing threads with actor *c*. According to our approach and the timestamps in Asterisk dataset, we decomposed the *P*-period into eight *P*-slices. According to Definitions 7 and 8, we got eight *P*-subgraphs and seven *S*-subgraphs.

Second, we gathered a dataset from Twitter via Twitter API from December 31, 2015, to January 06, 2016. The collected dataset is the data of hashtag #EndTaizSiege (14,944 actors and 46,552 connections) that comprises a big connected component (containing 84% of actors), singletons (14%), and smaller components (2%). We worked with the biggest component because that our goal is to evaluate the attractiveness of actors; hence, we focus on the biggest component which is considered as a single interaction domain for actors [3]. Applying our approach leads to decompose *P*-graph constructed from Twitter dataset into three *P*-subgraphs and two *S*-subgraphs based on three *P*-slices.

As a third example, we collected another dataset from Twitter from July 25 to July 30 in 2016. This Twitter dataset relates to the hashtag #coup_suffocates_Taiz (2241 actors and 4419 connections) that comprises a big connected component (containing 1418 actors). We divided the corresponding *P*-period into three *P*-slices. As a result, we obtained three *P*-subgraphs and two *S*-subgraphs.

**Fig. 6:**
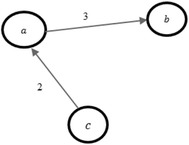
An example of graph representation for our Twitter datasets

The directed weighted *P*-graph of our collected Twitter datasets is constructed based on retweet activities so that actor *a* gets incoming connection from actor *b* if actor *b* retweeted a tweet of actor *a*. The weight of connection refers to the number of retweets activity between two connected actors. Figure 6 shows an example where actor *a* retweeted three tweets of actor *b* whereas the actor *c* retweeted two tweets of the actor *a*.

Boyd et al. [5] argued that retweet relation can be understood as a form of information diffusion and as a means of participating in an event in social media. Thus, we focus on retweet relation to evaluate our approach. Furthermore, we considered that retweet activity as attract an actor to become active in the community.

**Fig. 7:**
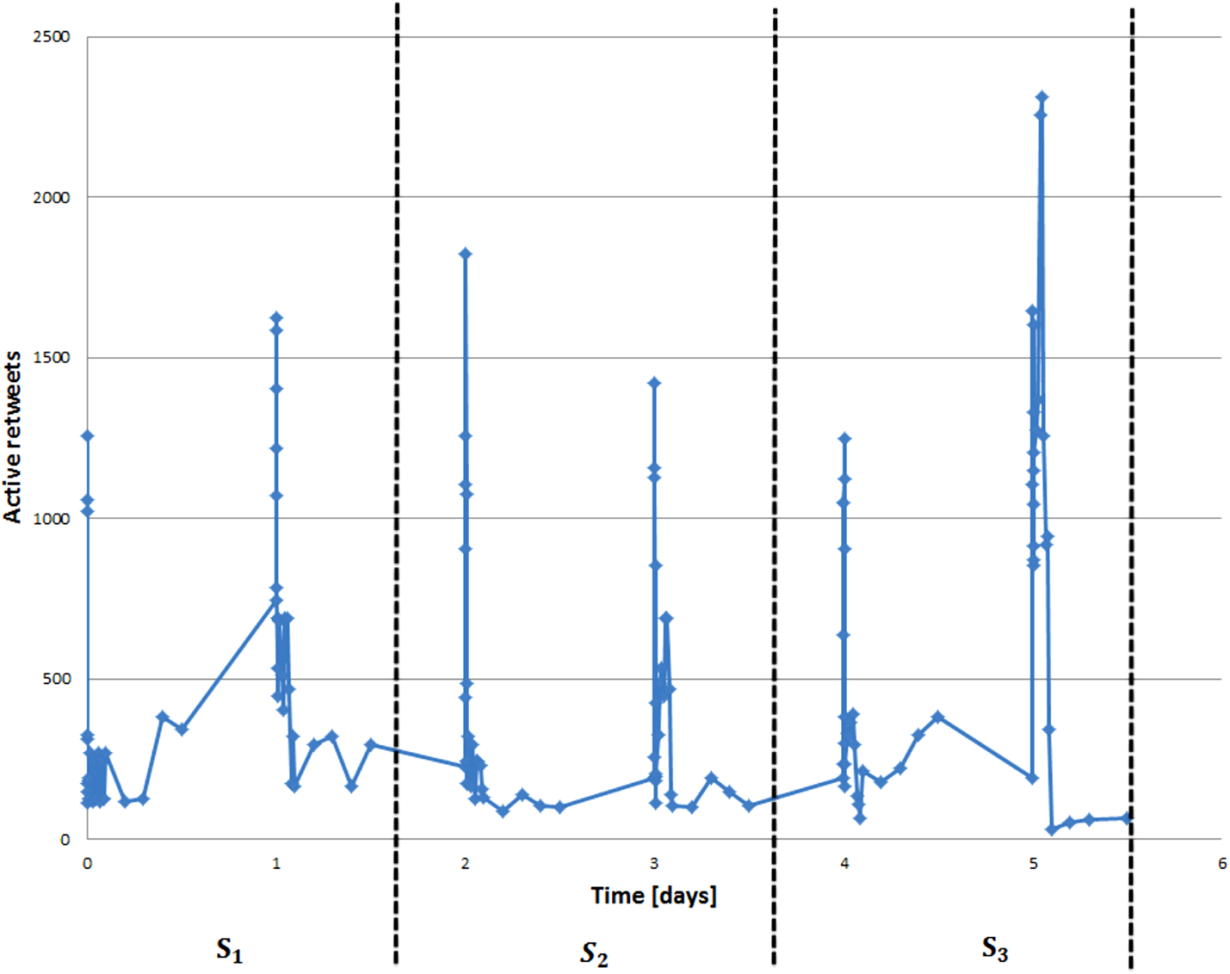
Retweet activities over time in Twitter dataset #EndTaizSieg

As a matter of fact, the time slicing does not depend on a specific predefined strategy but it has been estimated in accordance to the size of dataset using an equal window size for each slice. For instance, Fig. 7 shows how the *P*-period with Twitter dataset #EndTaizSiege has been decomposed into equal window size so that we get a fair division of the retweet activities for each time slice. (In our ongoing work, we try to find a general overall strategy for the time period decomposition).

### Experimental results

#### Asterisk

For Asterisk mailing lists dataset, we applied our *T* measure to verify whether our *T* measure can detect the influential actors. We got that *T* measure refers to the detection of influential actors in open source software developemnt projects as introduced by Zeini and Hoppe [25]. Actually, in open source projects, it is easy to find out the role of a community member because of the openness of the community archive including the full email communication and all code modifications. Hence, the positions of the actors in Asterisk dataset are well known (e.g. Kevin P. Fleming is a senior software engineer). Table 2 shows the top 10 actors with respect to *T*, degree, and betweenness measures.

**Table 2:** Top influential actors according to different influence measures over Asterisk dataset

Rank	T	Degree	Betweenness
1	Kevin P. Fleming	Kevin P. Fleming	Kevin P. Fleming
2	Tilghman Lesher	Olle E. Johansson	Olle E. Johansson
3	Tzafrir Cohen	Tzafrir Cohen	Tilghman Lesher
4	Russell Bryant	Tilghman Lesher	Tzafrir Cohen
5	Olle E. Johansson	Russell Bryant	Russell Bryant
6	Steven Critchfield	Steven Critchfield	Steven Critchfield
7	Eric Wieling	Tony Mountifield	Jared Smith
8	Jared Smith	Jared Smith	Tony Mountifield
9	Steve Totaro	Eric Wieling	Steve Totaro
10	Steve Murphy	Anton Vazir	Eric Wieling

To study the relation between *T* measure and other influence measures in Asterisk dataset, we used Spearman’s rank correlation coefficient $\rho$. Table 3 shows the different values of rank correlation. We notice that the significant correlation between *T* measure and other influence measures is relatively high. Thus, we can conclude that the attractors have also high values of other influence measures.

**Table 3:** Spearman’s rank correlation coefficient over Asterisk dataset

	T	Degree	Betweenness	Closenness	Eigenvalue
T	–	0.643	0.6930	0.551	0.574
Degree	–	–	0.869	0.864	0.910
Betweenness	–	–	–	0.668	0.716
Closeness	–	–	–	–	0.986
Eigenvalue	–	–	–	–	–

#### Twitter

For our Twitter datasets #EndTaizSiege and #coup_suffocates_Taiz, we investigate the relation between *T* measure and standard measures by Spearman’s rank correlation coefficient $\rho$. The results are shown in Tables 4 and 5.

- The rank correlation between indegree (retweets number) measure and number of followers is very low ($\rho$ = 0.08). This goes along with the findings of [3]. Thus, we can state that the popularity of actors in terms of the number of followers is not an important factor that affects retweet activities in Twitter.
- Furthermore, we found that the rank correlation between T and indegree (retweets number) measures is strong ($\rho$ = 0.6) and consequently, the correlation with the number of followers is low. This is reasonable since the *T* measure incorporates the indegree. However, in contrast to the indegree the *T* measure emphasizes attraction of new actors by not counting relations to actors who are already active in the community. This explains that these two measures are not more strongly correlated.
- Furthermore, we notice that the rank correlation between *T* and authority measures is high ($\rho$ = 0.5) but not as high as the correlation between the authority measure and indegree, which leads to the conclusion that the *T* measure also detects influential actors as the tradtional measures, but puts different emphasis on the attractors.

**Table 4:** Spearman’s rank correlation coefficient over Twitter dataset #EndTaizSiege

	Followers	T	Indegree	Outdegree	Betweenness	Hub	Authoritiy
Followers	–	0.1057	0.0805	0.0383	0.0871	0.0206	0.0780
T	–	–	0.6149	0.0027	0.5543	0.0013	0.4579
Indegree	–	–	–	−0.2600	0.6221	−0.2409	0.7555
Outdegree	–	–	–	–	0.3030	0.7298	0.2572
Betweenness	–	–	–	–	–	0.2464	0.4604
Hub	–	–	–	–	–	–	0.0916
Authority	–	–	–	–	–	–	–

**Table 5:** Spearman’s rank correlation coefficient over Twitter dataset #coup_suffocates_Taiz

	Followers	*T*	Indegree	Outdegree	Betweenness	Hub	Authoritiy
Followers	–	0.0921	0.0783	0.197	0.0815	0.0201	0.0639
*T*	–	–	0.6273	−0.1657	0.4231	−0.1485	0.4859
Indegree	–	–	–	−0.4345	0.4865	−0.4325	0.8035
Outdegree	–	–	–	–	0.2694	0.7878	0.0138
Betweenness	–	–	–	–	–	0.2169	0.3796
Hub	–	–	–	–	–	–	−0.1279
Authority	–	–	–	–	–	–	–

Tables 6 and 7 show also the correlation by Kendall’s rank correlation coefficient. The results shown here support our results which were investigated by Spearman’s rank correlation coefficient.

**Table 6:** Kendall’s tau rank correlation coefficient over Twitter dataset #EndTaizSiege

	Followers	*T*	Indegree	Outdegree	Betweenness	Hub	Authoritiy
Followers	–	0.0978	0.0612	0.0391	0.0773	0.0321	0.0562
*T*	–	–	0.5956	0.0015	0.5401	0.0028	0.4132
Indegree	–	–	–	−0.2361	0.5980	−0.1812	0.6823
Outdegree	–	–	–	–	0.2757	0.6077	0.3221
Betweenness	–	–	–	–	–	0.1944	0.4123
Hub	–	–	–	–	–	–	0.1088
Authority	–	–	–	–	–	–	–

**Table 7:** Kendall’s tau rank correlation coefficient over Twitter dataset #coup_suffocates_Taiz

	Followers	*T*	Indegree	Outdegree	Betweenness	Hub	Authoritiy
Followers	–	0.0671	0.0583	–0.0013	0.0515	–0.0088	0.0458
*T*	–	–	0.5993	–0.1466	0.4098	–0.1204	0.4257
Indegree	–	–	–	–0.3752	0.4605	–0.3433	0.7090
Outdegree	–	–	–	–	0.2408	0.6630	0.1383
Betweenness	–	–	–	–	–	0.1777	0.3325
Hub	–	–	–	–	–	–	–0.0477
Authority	–	–	–	–	–	–	–

Tables 8 and 9 show the description of the top influential actors in the Twitter datasets #EndTaizSiege and #coup_suffocates_Taiz with respect to T, indegree, and betweenness measures. The question mark in the table fields refers to an actor who is not a well-known influential actor within the community. We notice here how our *T* measure refers to the well-known influential actors within the community, or to the famous news accounts. Unlike other measures, the top ten influential actors with respect to *T* measure are well-known within the community. In our case, the well-known actors have been recognized based on a local expertise, where they are the most renowned actors in the field of human rights and politics who are continually traded their names in the newspapers and news concerning the current situation in Taiz city in Yemen. Their names have not been mentioned explicitly to protect their privacy.

**Fig. 8:**
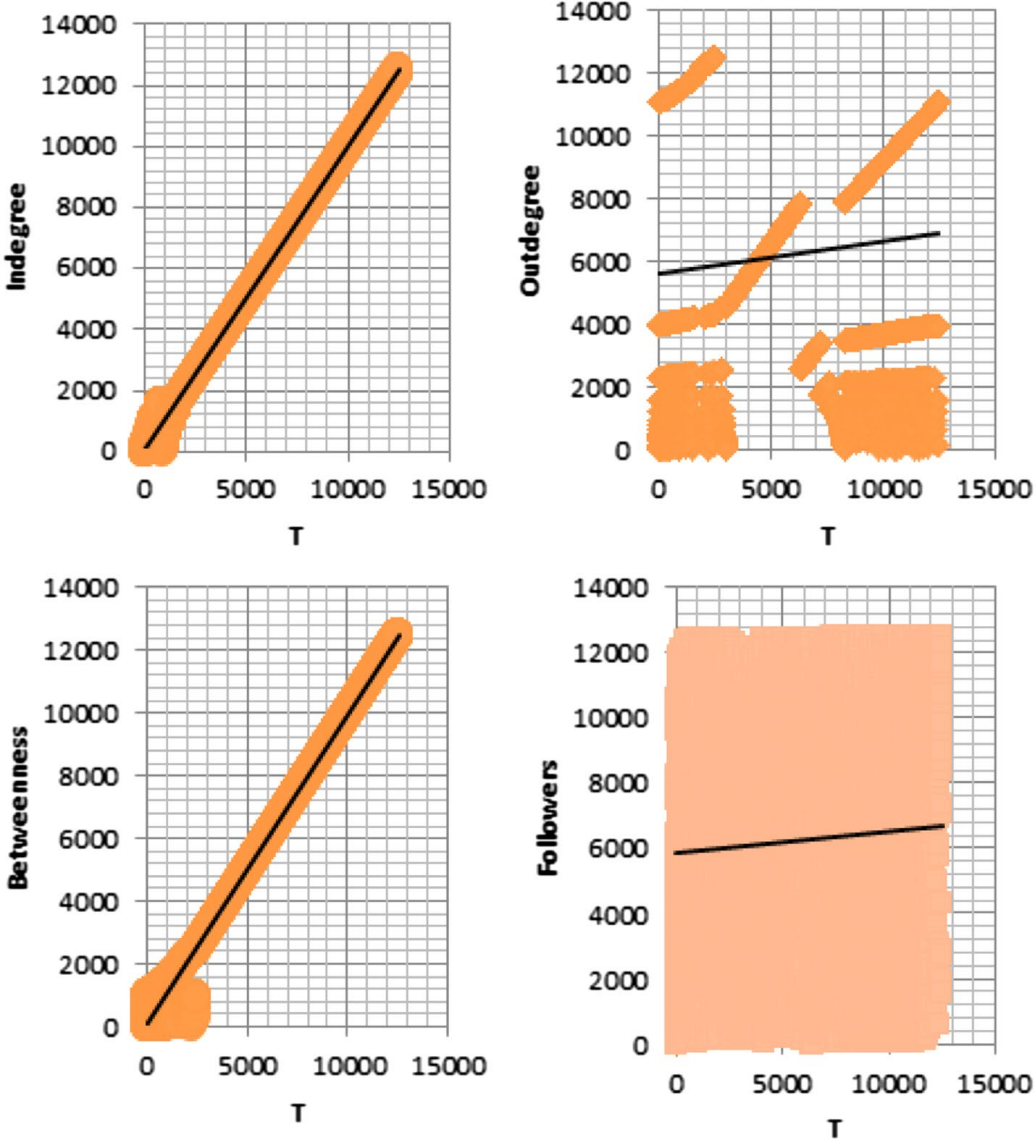
Distribution of *T* measure along with other standard measures over Twitter dataset #EndTaizSiege

Furthermore, we can note how the *T* measure is correlated with other standard measures from Fig. 8 that shows the distribution of *T* measure along with followers number, indegree, outdegree, and betweenness over the Twitter dataset. Figure 8 supports the results that were presented based on Spearman’s and Kendall’s rank correlation coefficient.

**Table 8:** Description of top influential actors according to different influence measures in Twitter dataset #EndTaizSieg

Rank	Description		
	*T*	Indegree	Betweenness
1	News account N1	News account N1	?
2	Journalist J1	Journalist J1	?
3	TV announcer T1	TV announcer T1	?
4	Television reporter R1	Journalist J3	Journalist J2
5	Human rights activist H1	Human rights activist H1	?
6	Human rights activist H2	News account N2	?
7	News account N2	Human rights activist H2	Human rights activist H3
8	Political activist P1	?	TV announcer T1
9	Journalist J2	Political activist P1	News account N1
10	Political activist P2	?	?

**Table 9:** Description of top influential actors according to different influence measures in Twitter dataset #coup_suffocates_Taiz

Rank	Description		
	*T*	Indegree	Betweenness
1	Journalist 1	Political activist	Journalist 1
2	Political activist	Journalist 1	Human rights activist
3	Joutnalist 3	Journalist 2	Journalist 2
4	News account 1	Joutnalist 3	?
5	Journalist 2	News account 1	?
6	Journalist 4	Human rights activist	?
7	Human rights activist	Politician	Joutnalist 3
8	?	?	?
9	Politician	?	?
10	News account 2	News account 2	?

## Information diffusion and *T* measure

To assess how well the *T* measure is suited to uncover influential actors with respect to information diffusion, we simulate the diffusion of information originating from a small seed set of nodes through the Twitter networks using the well-known independent cascade (IC) model [19]. To compare the performance of actors sets selected by the *T* measure with other influence measures, we selected sets of top actors based on the *T* measure and sets identified by measures that are known to be good heuristics for seed set selection, namely degree and betweenness centrality [26].
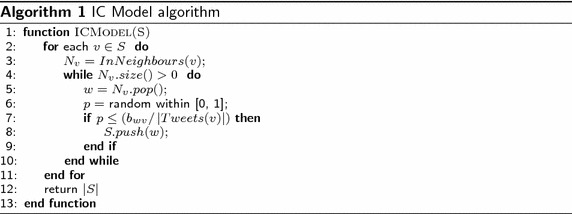


The IC model is an information diffusion model where the information flows over the network through cascade. Actors in the IC model can have two states, either active or inactive. Active means the actor is influenced by the information, and inactive means the actor is not influenced. The IC model calculation starts with an initial set of activated actors. In step *t*, an actor *a* will get a single chance to activate each currently inactive neighbor *b*. Actually, the activation process depends on the propagation probability *P* of the actors connection. The propagation probability *P* of a connection is the probability by which an actor can influence the other actors. In Twitter, we have proposed that actor *a* is influenced by actor *b* if he/she retweeted from actor *b* in proportion to the tweets number of actor *b*. So, the propagation probability *P* on IC model is based in our Twitter dataset on the connection weight divided by tweets number of target actor. The reason why we use the IC model instead of the LT model is that the linear threshold model is receiver oriented. This means an actor becomes active if a certain fraction of its neighbors are active. This does not account for our purpose where we want to find strong attractors who are likely to attract others. The IC model is sender oriented, and thus, is better suited to simulate attraction processes.

Algorithm 1 shows the pseudo code of IC model simulator which takes the seed set *S* as a parameter, and then evaluates the activated actors for the each actor *v* in the set *S*. Finally, it returns the total number of activated actors by whole actors in the set *S*.

### Simulation of attraction processes with time-respecting paths

In addition to the statistical comparison between the *T* measure and other standard network measures, we also report results based on simulated attraction processes. To do so, we adapt the IC model that is known to simulate the diffusion of information through a network as described above. Information diffusion and attraction processes have some commonalities but differ in various aspects. In traditional information diffusion models such as the IC model, the network is usually considered as stable in the sense that the set of nodes and the set of edges do not change over time. However, the nodes changes their states "inactive” and "active” during the information diffusion process. Attraction, as it is studied in this paper, is similar in the sense that actors who are not part of the community (i.e. do not have contributed a tweet) are inactive while others are considered as active. On the other hand, the original IC model does not account for the fact that the network grows when new actors become attracted to the community. Thus, the IC model was adapted to take into account the creation times of the edges. These time-varying networks have special characteristics regarding reachability of node pairs since a walk on the graph can only take edges with increasing timestamp, which is known as the time-respecting property (see [27, 28]). In this aspect, we added a new activation rule to the IC model which is: the actor who is activated in time *t* cannot activate those actors who have been linked with him/her before the time *t*. To explain this activation rule in more detail, we define the following terms:

#### **Definition 12**

(*Pathtime*) The path time of each link in the network is the *P*-slice number in which this link has been created.

#### **Definition 13**

(*Activation time*) The activation time of each activated actor is the path time of the link by which this actor has been activated.

Now, we can state that the actor *a* cannot activate the actor *b* if the link from *b* to *a* has a path time later than the activation time of the actor *a*.

Using this activiation rule, the simulation can be interpreted as an attraction process where actors who are already part of the communities can attract others only if their activity starts after the activator has become active.

Previous studies [1] have shown that a seed selection strategy based on indegree yields similar results as a selection strategy based on the *T* measure. This is also expected with respect to the high correlation between these two measures. However, the benefit of the *T* measure that distinguishes it from other measures is that time is explicitly taken into account. The experimental results in the next section support the assumption that the *T* measure can identify important attractors in time-varying networks while it boils down to indegree if time is neglected.

### Experimental results

**Fig. 9:**
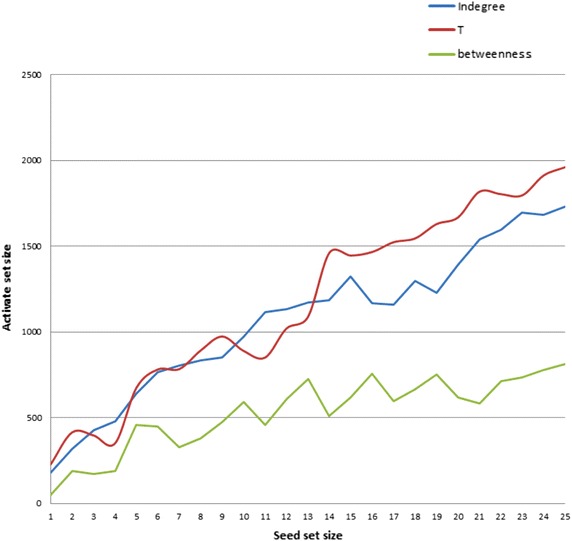
IC model under time-respecting paths with different influence measures over Twitter dataset #EndTaizSiege

Here, we considered the dataset #EndTaizSiege which is related to an organized event in Yemen. Hence, we got a highly connected component that is suitable for the application of our approach which is basically aimed to identify those actors who contribute to attract others to participate in a specific organized event. We simulated the information diffusion based on the IC model with time-respecting paths for seed sets of sizes $n = 1 \ldots 25$ which are generated from different influence measures. Figure 9 shows the results of applying the IC model to seeds generated from T, indegree, and betweenness measures. We notice that the T measure yields the best performance in information diffusion under the IC model with time-respecting paths for the seed sizes bigger than 13. Additionally, we statistically verified the results of simulation for each seed set using* T* test. In case of n (n > 13), the differences among *T* and indegree measures are significant. For example, results for the seed set 14 show that there is a significant difference in the score of *T* measure $(M = 1462.1,\,{\it SD} = 85.3802 \,{\text{conditions}}; \,\,t(19) = 14.4854,\,P = 0.0000)$. Table 10 presents the relevant descriptive statistics.

**Table 10:** *T* test verification for simulation results in case of seed sizes* n* (*n* > 13) among *T* and indegree measures in the dataset #EndTaizSiege

Seed size	* t*	* df*	Sig. (2-tailed)	95% confidence interval		Mean difference	Mean	Std. deviation
				Lower	Upper			
14	14.4854	19	0.0000	1422.1408	1502.0592	276.55	1462.1	85.3802
15	10.5787	19	0.0000	1415.4421	1476.6579	154.7	1446.05	65.3996
16	14.7604	19	0.0000	1424.0960	1509.2040	300.1	1466.65	90.9247
17	18.2705	19	0.0000	1482.1069	1565.4931	363.95	1523.8	89.0852
18	11.6923	19	0.0000	1501.8185	1590.4815	247.65	1546.15	94.7225
19	26.9261	19	0.0000	1598.1139	1660.4861	401.2	1629.3	66.6350
20	16.3709	19	0.0000	1632.5976	1702.9024	274.95	1667.75	75.1097
21	17.4834	19	0.0000	1784.6586	1850.7414	276	1817.7	70.5990
22	12.2143	19	0.0000	1768.7146	1840.0854	208.25	1804.4	76.2485
23	6.8975	19	0.0000	1766.6357	1827.2643	99.9	1796.95	64.7720
24	17.6846	19	0.0000	1885.3439	1939.6561	229.45	1912.5	58.0240
25	17.5075	19	0.0000	1933.0513	1987.9487	229.6	1960.5	58.6493

Here, we considered the dataset #EndTaizSiege which is related to an organized event in Yemen. Hence, we got a highly connected component that is suitable for the application of our approach which is basically aimed to identify those actors who contribute to attract others to participate in a specific organized event. We simulated the information diffusion based on the IC model with time-respecting paths for seed sets of sizes $n = 1 \ldots 25$ which are generated from different influence measures. Figure 9 shows the results of applying the IC model to seeds generated from* T*, indegree, and betweenness measures. We notice that the T measure yields the best performance in information diffusion under the IC model with time-respecting paths for the seed sizes bigger than 13. Additionally, we statistically verified the results of simulation for each seed set using* T* test. In case of* n* (*n* > 13), the differences among *T* and indegree measures are significant. For example, results for the seed set 14 show that there is a significant difference in the score of *T* measure $(M = 1462.1, SD = 85.3802 \, {\,\text{conditions}};\, t (19) = 14.4854, P = 0.0000)$. Table 10 presents the relevant descriptive statistics.

**Fig. 10:**
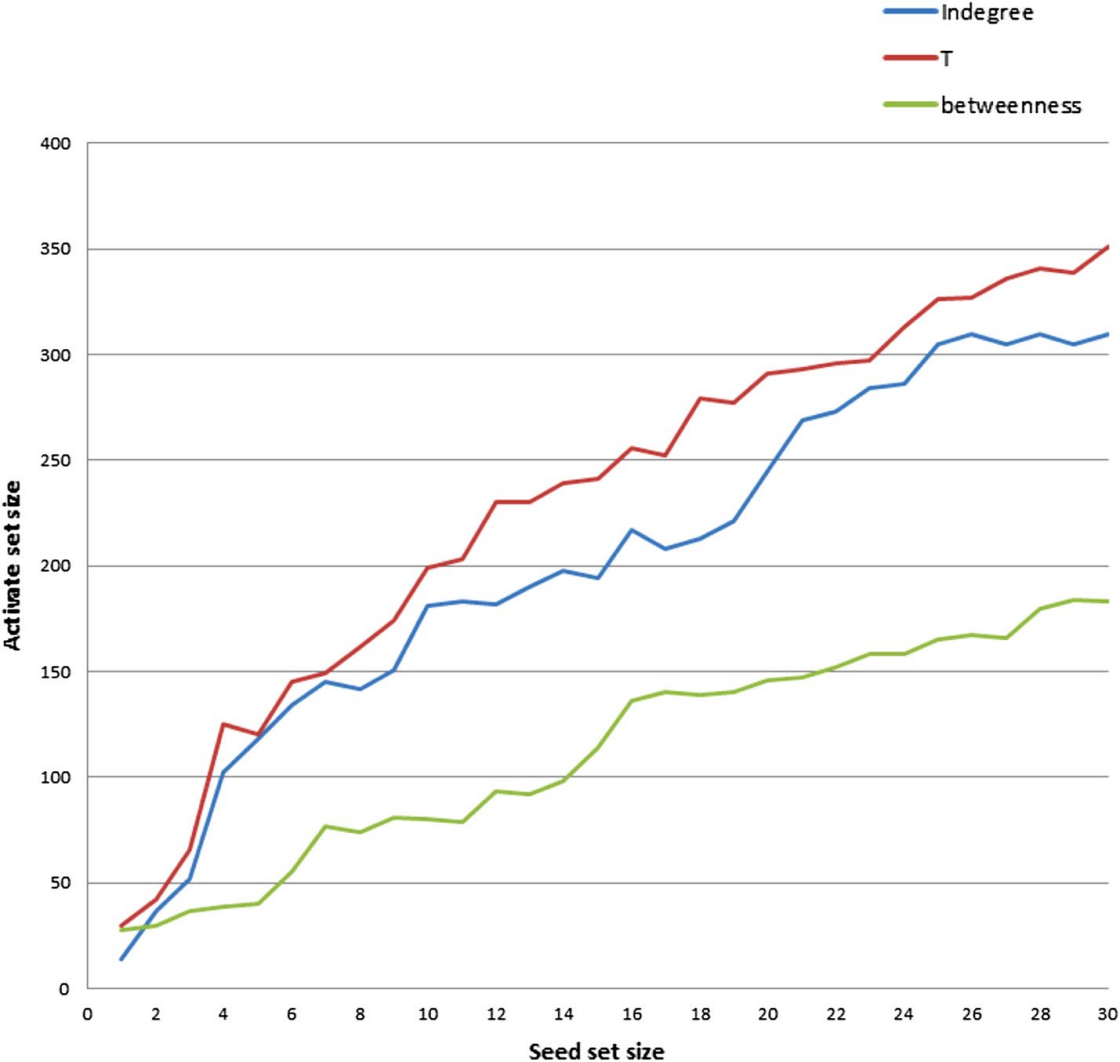
IC model under time-respecting paths with different influence measures over Twitter dataset #coup_suffocates_Taiz

Furthermore, we consider the dataset #coup_suffocates_Taiz. We simulated here for seed sets of sizes $n = 1 \ldots 30$ which are generated from different influence measures. Figure 10 shows the results of applying the IC model to seeds generated from T, indegree, and betweenness measures. We notice that the T measure yields the best performance in information diffusion under the IC model with time-respecting paths for the seed sizes bigger than 7. Additionally, we statistically verified the results of simulation for each seed set using* T* test. In case of* n* (*n* > 7), the differences among *T* and indegree measures are significant. For example, results for the seed set 8 show that there is a significant difference in the score of *T* measure $(M = 162, SD = 16.946 \, {\,\text{conditions}};\, t (19) = 3.272, P = 0.00)$. Table 11 presents the relevant descriptive statistics.

**Table 11:** *T* test verification for simulation results in case of seed sizes* n* (*n* > 7) among *T* and indegree measures in the dataset #coup_suffocates_Taiz

Seed size	* t*	*df*	Sig. (2-tailed)	95% confidence interval		Mean difference	Mean	Std. deviation
				Lower	Upper			
8	3.272474738	19	0.004	154.0691524	169.9308476	12.4	162	16.94573382
9	8.159694936	19	0.000	167.6259751	179.3740249	22.9	173.5	12.5509488
10	5.02467484	19	0.000	191.1521152	206.1478848	18	198.65	16.02062815
11	3.22614144	19	0.004	190.3718072	202.8281928	9.6	196.6	13.30769465
12	21.28977767	19	0.000	222.9571772	231.9428228	45.7	227.45	9.599753286
13	11.30200169	19	0.000	219.9183376	232.3816624	33.65	226.15	13.31510816
14	13.11109148	19	0.000	226.1143374	236.9856626	34.05	231.55	11.61430606
15	12.14162861	19	0.000	230.4304495	243.6695505	38.4	237.05	14.14390328
16	8.499171278	19	0.000	246.3088375	265.5911625	39.15	255.95	20.60014052
17	10.90348517	19	0.000	245.934543	258.565457	32.9	252.25	13.49415078
18	19.29415746	19	0.000	272.7273155	285.9726845	61.05	279.35	14.15059976
19	20.77030073	19	0.000	269.3351504	279.7648496	51.75	274.55	11.14249807
20	13.09099585	19	0.000	284.2110899	298.0889101	43.4	291.15	14.82627469
21	7.505556897	19	0.000	283.7953814	293.3046186	17.05	288.55	10.15912864
22	2.617290607	19	0.002	285.5219614	295.6780386	6.35	290.6	10.85017584
23	1.160344906	19	0.003	285.6721647	302.6278353	4.7	294.15	18.11447517
24	5.3893686	19	0.000	306.6255244	319.6744756	16.8	313.15	13.94075812
25	0.946308607	19	0.004	307.8905455	322.7094545	3.35	315.3	15.83168043
26	3.909066253	19	0.001	313.7701177	322.2298823	7.9	318	9.037931762
27	8.922128329	19	0.000	325.3910277	333.1089723	16.45	329.25	8.245413398
28	11.85052393	19	0.000	336.3281838	346.2718162	28.15	341.3	10.62321192
29	9.295528476	19	0.000	333.9635987	344.6364013	23.7	339.3	11.40221585
30	13.14909142	19	0.000	345.7835107	356.4164893	33.4	351.1	11.35967012

## Conclusion

In this paper, we introduced a new approach to detect influential actors based on a new type of influence. Influential actors who are detected by our approach are those actors whose tweets spawn many retweets in a way that leads to an increase in the size of social network. We presented through experiment results how our proposed measure *T* referred to the influential actors in Asterisk and Twitter datasets. Furthermore, we introduced the relation between *T* measure and other influence measures using Spearman’s rank correlation. Finally, we showed through experiment and statistical tests that the best performance has been yielded by *T* measure in maximization of influence problem when we took the time into account.

Our current work in extending and improving this approach focuses on a differentiation of the role of the actors and different types of communication networks based on the *T* measure. As well as, we plan to describe our approach on multilayer networks. Furthermore, we are going to study an efficient general strategy to define the size of *p*-slice depending on the premise: the* p*-slice is the time that the most tweets get the most of their retweets. Moreover, we intend to study the role of time slicing in making *T* measure far better than existing measures.

## References

[1] Qasem Z, Jansen M, Hecking T, Hoppe HU. On the detection of influential actors in social media. In: 11th international conference on signal-image technology and internet-based systems. Washington, DC, USA: IEEE Computer Society. 2015. p. 421–27.

[2] Leavitt A, Burchard E, Fisher D, Gilbert S (2009). The influentials: new approaches for analyzing influence on twitter. Web Ecol Proj.

[3] Cha M, Haddadi H, Benevenuto F, Gummadi PK (2010). Measuring user influence in twitter: The million follower fallacy. International conference on weblogs and social media.. ICWSM.

[4] Azaza L, Kirgizov S, Savonnet M, Faiz R. Influence assessment in Twitter Multi-Relational Network. In: 2015 11th international conference on signal-image technology and internet-based systems (SITIS). Washington, DC: IEEE; 2015. p. 436–43.

[5] Boyd D, Golder S, Lotan G. Tweet, tweet, retweet: Conversational aspects of retweeting on twitter. In: Hawaii international conference on system sciences. Honolulu: IEEE; 2010.

[6] Yamaguchi Y, Takahashi T, Amagasa T, Kitagawa H (2010). Turank: Twitter user ranking based on user–tweet graph analysis. InInternational Conference on Web Information Systems Engineering.

[7] Weng J, Lim EP, Jiang J, He Q (2010). Twitterrank: finding topic-sensitive influential twitterers. InProceedings of the third ACM international conference on Web search and data mining.

[8] Anagnostopoulos A, Kumar R, Mahdian M (2008). Influence and correlation in social networks. In: Proceedings of the 14th ACM SIGKDD international conference on Knowledge discovery and data mining.

[9] Crandall D, Cosley D, Huttenlocher D, Kleinberg J, Suri S (2008). Feedback effects between similarity and social influence in online communities. In: Proceedings of the 14th ACM SIGKDD international conference on knowledge discovery and data mining.

[10] Liu L, Tang J, Han J, Jiang M, Yang S (2010). Mining topic-level influence in heterogeneous networks. In: Proceedings of the 19th ACM international conference on information and knowledge managemen.

[11] Rogers EM (2003). Diffusion of innovations.

[12] Gruhl D, Guha R, Liben-Nowell D, Tomkins A (2004). Information diffusion through blogspace. In: Proceedings of the 13th international conference on World Wide Web.

[13] Yang J, Counts S (2010). Predicting the speed, scale, and range of information diffusion in twitter. International conference on weblogs and social media. ICWSM.

[14] Vallet J, Kirchner H, Pinaud B, Melançon G. A visual analytics approach to compare propagation models in social networks. arXiv: arXiv:1504.02612. 2015.

[15] Goldenberg J, Libai B, Muller E (2001). Talk of the network: a complex systems look at the underlying process of word-of-mouth. Mark Lett.

[16] Goldenberg J, Libai B, Muller E (2001). Using complex systems analysis to advance marketing theory development: modeling heterogeneity effects on new product growth through stochastic cellular automata. Acad Mark Sci Rev.

[17] Granovetter M (1978). Threshold models of collective behavior. Am J Sociol.

[18] Chen W, Yuan Y, Zhang L (2010). Scalable influence maximization in social networks under the linear threshold model. In: 2010 IEEE international conference on data mining.

[19] Kempe D, Kleinberg J (2003). Maximizing the spread of influence through a social network. In: Proceedings of the ninth ACM SIGKDD international conference on knowledge discovery and data mining.

[20] Pei S, Muchnik L, Andrade Jr JS, Zheng Z, Makse HA (2014). Searching for superspreaders of information in real-world social media. Sci Rep.

[21] Kempe D, Kleinberg J, Tardos É (2005). Influential nodes in a diffusion model for social networks. Automata., Languages and Programming.

[22] Chen W, Wang Y, Yang S (2009). Efficient influence maximization in social networks. In: Proceedings of the 15th ACM SIGKDD international conference on knowledge discovery and data mining.

[23] Pei S, Makse HA (2013). Spreading dynamics in complex networks. J Stat Mech.

[24] Morone F, Makse HA (2015). Influence maximization in complex networks through optimal percolation. Nature.

[25] Zeini S, Hoppe U. Community Detection alsAnsatz zur Identifikation von Innovatoren in Sozialen Netzwerken. In: Klaus Meißner, Martin Engelien: Gemeinschaften in Neuen Medien (GeNeMe). Tagungsband. TU Dresden 2011, ISBN 978-3-942710-35-0. 2010. p. 131–40.

[26] Mochalova A, Nanopoulos A (2013). On the role of centrality in information diffusion in social networks. In: 21st European conference on information systems.

[27] Holme P, Saramäki J (2012). Temporal networks. Phys Rep.

[28] Casteigts A, Flocchini P, Quattrociocchi W, Santoro N (2011). Time-varying graphs and dynamic networks. In: International conference on Ad-Hoc networks and wireless.

